# Topolectrical Circuit Correspondence Design of Polyacetylene

**DOI:** 10.1038/s41598-023-48278-z

**Published:** 2023-11-27

**Authors:** Majid Reza Albooyeh, Ali Sadeghi, Seyed Majid Mohseni

**Affiliations:** Department of Physics, Shahid Beheshti University, Tehran, 19839-69411 Iran

**Keywords:** Surfaces, interfaces and thin films, Topological matter

## Abstract

In *cis* and *trans* geometrical configurations of the polyacetylene molecule, one-dimensional chain is constructed by attaching a number of identical –HC=CH– units one-by-one. We attach as many units as required to obtain the chain of the desired length. In case of a very long polyacetylene chain, which is practically considered infinite in length, a periodic unit is defined, so that its band structure would be calculable. Then, the electronic properties and topological properties of the chain can be predicted. Since experimental synthesis of single-layer polyacetylene chain has lots of limitations, in an alternative approach, emulation of a tight-binding model is used to describe the electron transfer in polyacetylene polymer chain. In case of either synthesis or testing the polyacetylene molecule, it is necessary to improvise a one-to-one correspondence between polyacetylene polymer and topological circuit, which is introduced for the first time in the present study. To this aim, the outputs of density functional theory calculations alongside with the calculations based on the physical chemistry formalisms are used. Here, we observed that the electronic response of the circuit is topologically sustained at frequencies where the coupling was pre-determined via high precision quantum system equivalent topolectrical circuit, as an alternative classical system, to study electron transfer of *trans*-polyacetylene polymer quantum chain by the precision of one-electron.

## Introduction

Edge-state of quantum materials was first discovered via finding of soliton formation in long-chain of polyenes^1^, and followed by studying quantized Hall resistance in two-dimensional electron gas in metal-oxide semiconductor field-effect transistor^2^. The last two decades was a glorious time of this field when attention was made on topological systems^3,4^ and also opening research field in photonics, cold-atom, microwave, phonon and magnon systems^5–9^.

Shortly after introducing the *Berry* phase in quantum systems, *J. H. Hannay* determined that similar geometrical effects can be described using classical mechanics’ Hamiltonian equation (Supplementary information—Sect. 1)^10^. In other words, the topological states that are studied in quantum systems, could be studied in the classical systems as well. Considering the fact that the “*Berry-phase*” is as an index of showing how the material has topological properties in quantum terms^10^, one can tell the created “frequency-gap” in the phonon spectrum of a simple tensegrity structure (as a model for a classical meta-material with topological properties) could be considered as the classification index in classical terms as well. Then it is checked how the “frequency-gap” is correlated to the edge states. Previous analysis indicates the existence of newly known mechanical phases, which are topologically distinct. The protected edge states could be described by one-dimensional modeling, expressed in the form of SSH model (Su, Schrieffer, Heeger), for mapping of polymer’s equivalent electrical circuit (Supplementary information—Sect. 2–3)^11^.

The relation between the various parameters in non-self-consistent semi-empirical tight binding (TB) calculations and self-consistent density functional theory (DFT), relies on either TB energy expression or the short-range and transferable matrix elements^12^. Prediction of the energy gap and other parameters of interest related to the band structure is mapped in the tight-binding Hamiltonian system^12^. In order to screen out the Coulomb interaction, one can solve the Schrodinger-like “*Kohn–Sham*” equations for the valence electrons. By this simplified method, one can ignore the core electron potential in favor of the valence pseudopotential^12^. The *Kohn–Sham* approach over the past decades has led to the development of more than a hundred software packages (including Quantum Espresso) with default numerical implementations of DFT^13–17^. The wave function attributed to real atomic orbitals, results in the creation of artificial orbitals known as “*Kohn–Sham* orbitals”^13,16^. The DFT calculations have been very efficient for systems with a large number of electrons^18^.

Study of topological electrical circuits is a nascent and rapidly developing scope that analyzes the electronic structures of materials, based on the topological characteristics of their eigenstates^9^. This scope of science is inspired by extensive studies that were previously conducted in the field of dense matter physics, which used to focus on the relationship between topology and various phenomena^19^. Further, in the last decade, obtaining new findings resulted in extension of the concept of topology from solid state electronic structures to other structures with similar dynamics, such as photonic and acoustic systems^19,20^.

Due to the laboratory limitations of the synthesis and testing of polyacetylene polymer, it is necessary to construct an electrical circuit with the same topolectrical properties as polyacetylene polymer, which is the main discussed approach in the present study. Here, experimental monitoring of the polyacetylene’s topological behavior was obtained according to the Hamiltonian matrix of the circuit. Here, We introduce a one-dimensional (1D) model that illustrates this phenomenon in its simplest form and maps directly to the SSH model for the electronic excitations of polyacetylene ((CH2)n)^11^. Our analysis allows us to predict the existence of new topologically distinct bulk mechanical phases and to characterize the protected modes that occur on their boundary.

## Results

In this study, numerical values of ${t}_{1}$ and ${t}_{2}$ couplings were fitted to the band structures calculated using Quantum Espresso software^13^. It should be noted that the geometrically optimized bond lengths, are in absolute agreement with experimental values^21,22^. Using plane waves as the basis set for expanding the wave function, the QE package is suitable for the calculations of systems that are alternating periodic in all three dimensions. We set the kinetic energy cutoff 30 Ry. The self-consistent cycle is looped until the total energy converges, *i.e.* when the change in energy in two consequent cycles falls below 0.0001 atomic units (2.7 meV). Then, the numerical values of C_b_, C_a_, and L were obtained from Eq. (1). By using these values, the equivalent electrical circuit was designed and built according to the one-dimensional one-layer structure of polyacetylene polymer. In this regard, the correspondence of the performance of the constructed topolectrical circuit with the electrical properties of polyacetylene polymer was studied. The details of which are given below (Also see Supplementary information—Sect. 4).

### Outputs of Quantum Espresso software

**Figure 1:**
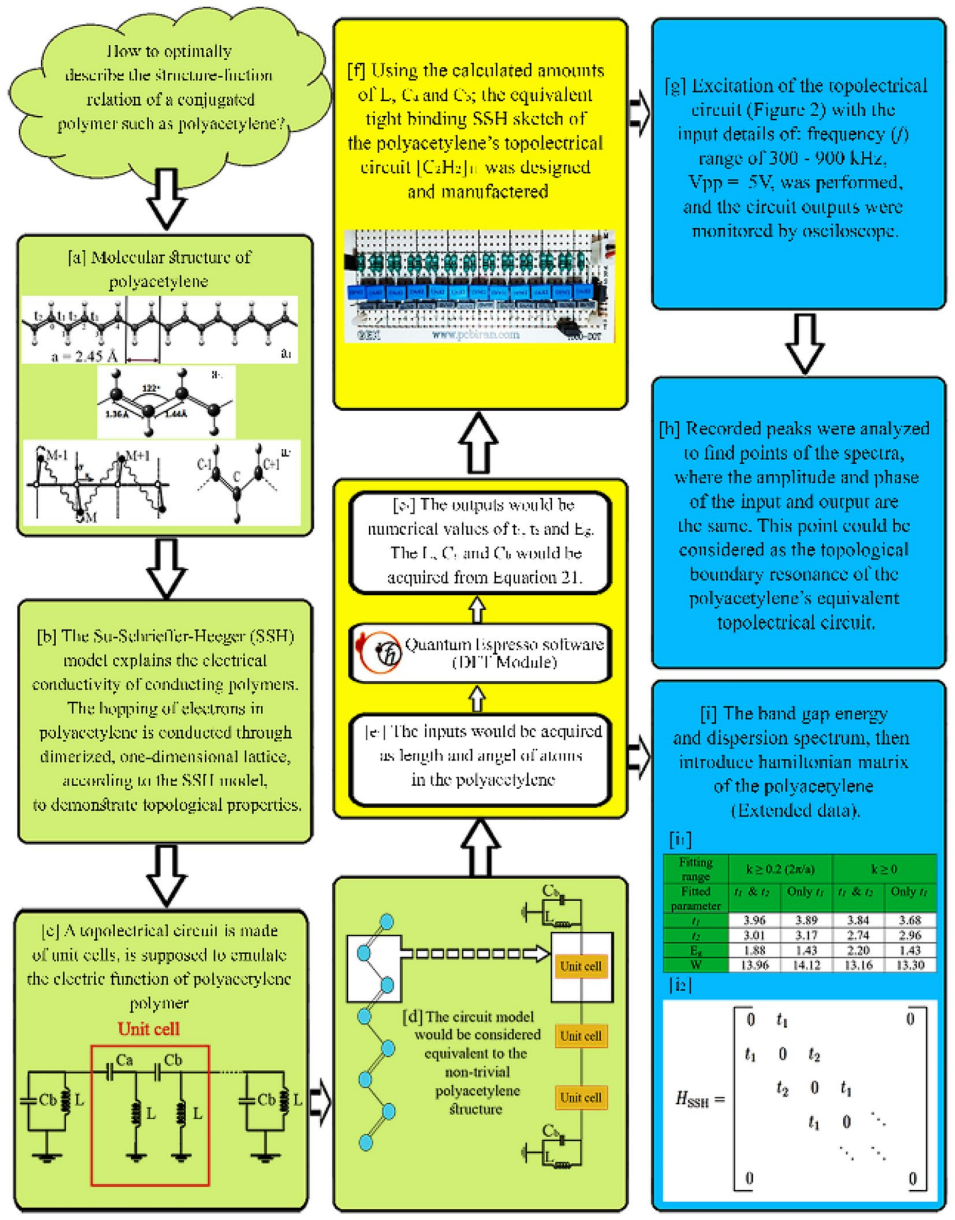
Visual outline of all steps performed in the present study. The green, yellow, and blue panels indicate the conceptual design, method, and results, respectively. (**a**_**1**_) *Trans* configuration of polyacetylene chain in one-dimensional periodic structure. The meaning of “a”, which is indicated in the figure, is the lattice constant with angstrom (Å) unit. (**a**_**2**_) Stable structure of polyacetylene molecule with *trans* configuration (zigzag), where the single and double bond lengths are 1.44 Å and 1.36 Å, respectively. The angle between three consecutive carbon atoms is 122 degrees. (**a**_**3**_) (Left side) is a part of the network of coupled gyrators, which is the classical equivalent (right side) of a part of the quantum chain of the polyacetylene polymer. (**b**) The SSH model describes how electron hopping occurs in a semi-finite chain of the conducting polymer polyacetylene. (**c**) Unit cell structure of the polyacetylene equivalent topolectrical circuit, as the building units of the circuit. (**d**) Schematic profile of the one-to-one correspondence of the constituent parts of the polymer and the equivalent topolectrical circuit of polyacetylene. (**e**_**1**_–**e**_**2**_) DFT module inputs and outputs of Quantum Espresso software. (**f**) Designing and building a topolectrical circuit, based on the outputs of Quantum Espresso software. (**g**) Excitation and recording conditions of the equivalent topolectrical circuit of polyacetylene. (**h**) Analysis of the recorded outputs of the topolectrical circuit, in search of the edge-effect. (**i**) Comprehensive characterization of the conductivity of polyacetylene polymer as a topological system, using dispersion spectrum. (**i**_**1**_) Electron’s hopping parameters, energy gap and bandwidth W = 2(t_1_ + t_2_), obtained from fitting the dispersion relation to DFT calculation data. The unit of all values is in electron volt (eV). (**i**_**2**_) The SSH model is a tight binding 1D chain with conjugating hopping parameters.

Using the hybrid B3LYP functional, available in Quantum Espresso software, the energy gap of polyacetylene in the *trans* configuration is 1.43 eV. The obtained band structure includes six energy bands. The five lowest energy bands are occupied by the 10 electrons of the unit cell. One electron for each hydrogen atom and 4 electrons for each carbon atom occupy the 2 s and 2p orbitals in the calculations. Since each unit cell has two hydrogen atoms and two carbon atoms, a total of 10 electrons per unit cell have been included in the calculations, which, including the upper and lower spins, finally fill the lower five states^23^. Energy dispersion in the last occupied band and the first unoccupied band, especially close to the edge of the Brillouin zone (i.e. $k=0.5 (\frac{2\pi }{a})$), where a direct energy gap with a width of 1.43 eV, is the most important result of DFT calculations. The parameters of the tight-binding model are determined by fitting this part of the band structure, which is explained in Fig. 1 (panel—i_1_).

**Figure 2:**
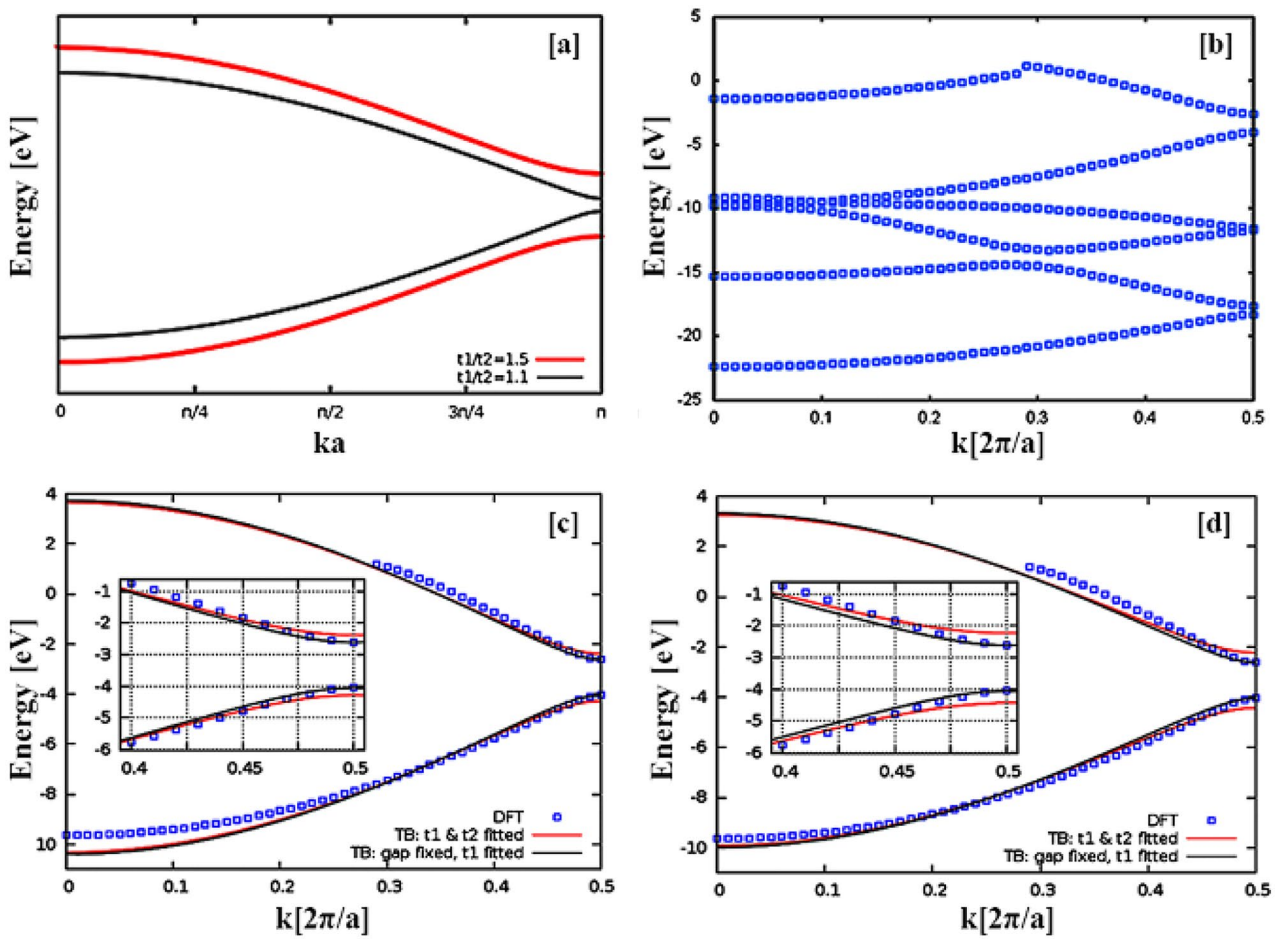
Dispersion spectrum of polyacetylene polymer. (**a**) the band structure of the polyacetylene chain in the tight-binding model for two different ratios of hopping parameters. In each case, the lower band (conduction) is occupied by electrons and the upper band (valence) is unoccupied. (**b**) the band structure of polyacetylene obtained from DFT calculations using the B3LYP function. Up to the fifth band (counting from bottom to top) is occupied by electrons and the sixth band is remained unoccupied. Only the highest two bands are used for fitting of the two-state tight-binding model. (**c**) Fitted tight-binding dispersion relation to the energy bands of the DFT framework. When both parameters (red solid line) or only one of them (black solid line) are fitted to the data in the interval (i.e. k ∈ [0.2,0.5] × 2π/a), the results would be the ones in the Fig. 1i1. (**d**) Like Panel c, the fitting is done, but to the entire data interval (*i.e.* k ∈ [0,0.5] × 2π/a). The fitting results are listed in Fig. 1i1.

The band structure for two different ratios of hopping parameters is shown in Fig. 2 (Panels c and d). The results of both fitting methods are shown in Fig. 2 (Panels c and d), and the fitted values are listed in Fig. 1i1. The numerical results are somewhat dependent on the fitting method and the intervals, which used to fit the data, but the pattern is consistent with the prediction of the tight-binding model.

### Experimental outputs resulting from the equivalent electric circuit

**Figure 3:**
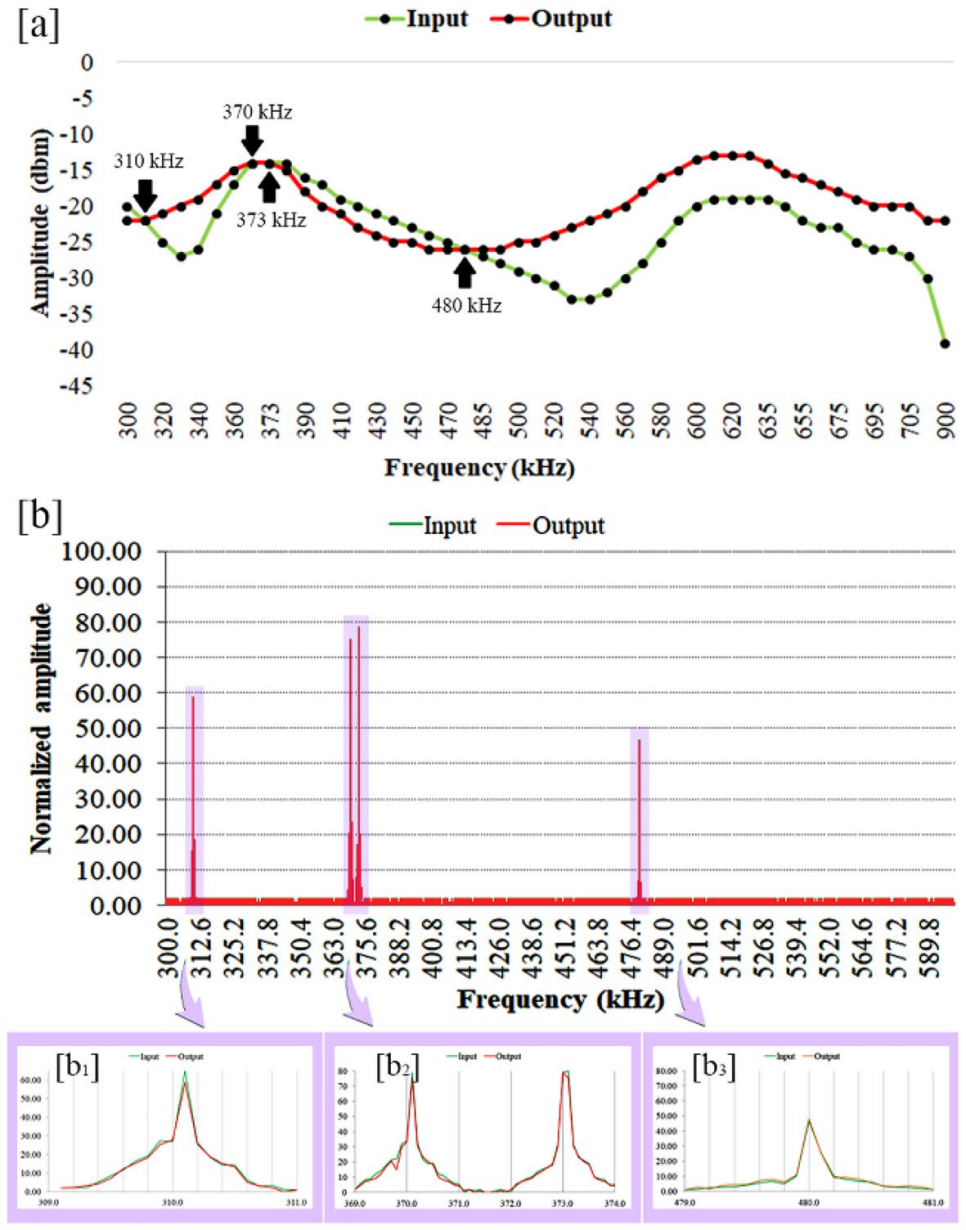
Amplitude and phase profiles obtained from input and output records taken with an (**a**) oscilloscope. (**b**) The input and output signals recorded with a spectrum analyzer and represented as normalized amplitude. Since the input and output amplitudes are completely matched, only the output peaks are demonstrated here. The peaks that emerge with the same amplitude are highlighted in b_1_ to b_3_ panels, respectively.

The topolectrical circuit, equivalent to polyacetylene, was stimulated with two topological and non-topological modes and the resulting outputs were recorded with an oscilloscope. By recording the inputs and outputs, it was shown that the input and output amplitudes were found to be equal at four frequencies (310, 370, 373 and 480 kHz). Further, at one out of four recording frequencies, it was observed that the input and output states were in the same phase in addition to being of the same amplitude (Fig. 3a).

In this way, at the recording frequency of 373 kHz, the absolute electrical conduction occurs in the equivalent polyacetylene polymer electrical circuit.

## Discussion

Unlike the energy of the ground state, the energy of the unoccupied states, and therefore the energy gap, may have a significant difference in reality and laboratory results. Many efforts have been made to solve these shortcomings, and nowadays so-called hybrid density functional have been designed to estimate the effects of electron exchange–correlation, which then impose more computational cost, have provided high-accuracy energy gap prediction.

Fortunately, hybrid DFT calculations that reproduce experimental results with high accuracy are nowadays feasible for even much more complex structures than polyacetylene^24^. Our results show that in the band structure of polyacetylene, obtained from DFT calculations with the B3LYP functional, only two higher energy bands (Fig. 2b) are used to fit the tight-binding two-state model. Note that, in contrast to the DFT calculations, only two bands are considered in the tight-binding model. In particular, for the highest energy band, the data-points from DFT calculations for k < 0.28 [2π/a] correspond to an energy band which is missing in the two-state tight-binding band structure. We therefore use only the segment k > 0.28 [2π/a] from the DFT band structure in order to fit the corresponding segment of the energy band of the tight-binding model. The fitting results, based on the two applied ranges (k ∈ [0.2,0.5] × 2π/a and k ∈ [0,0.5] × 2π/a) show that the obtained E_g_ (1.88 electron volts—Fig. 1il) is completely consistent with the prediction of experimental findings^23,25^, while the E_g_ obtained from Supplementary information—Eq. (17c) was consistent with the theoretical expectation^26^ (1.43 electron volts—Fig. 1il).

**Figure 4:**
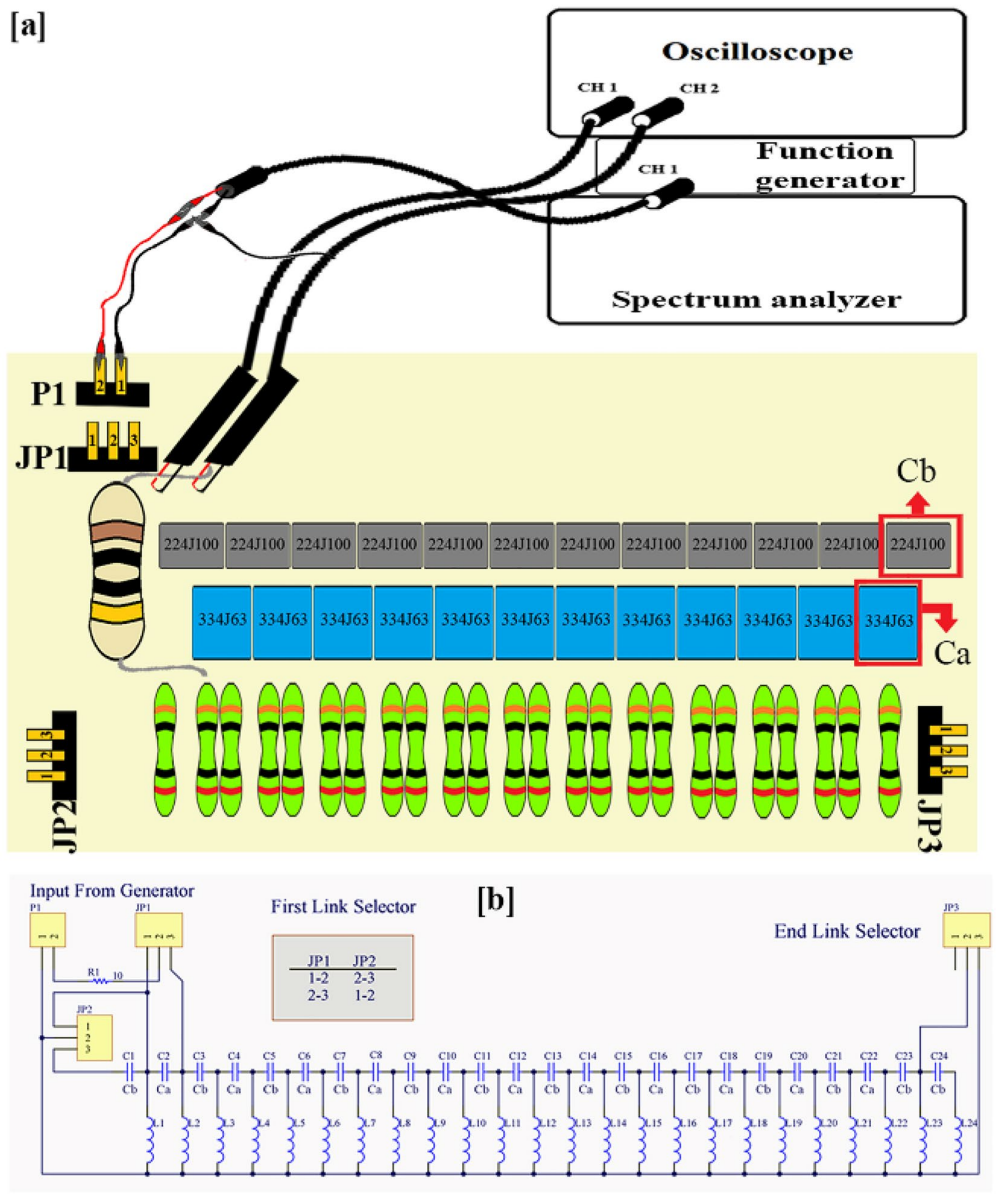
Experimental set-up for stimulating and recording the polyacetylene’s equivalent topolectric circuit. (**a**) The schematic profile of the set-up is demonstrated. The circuit was excited with function generator and its output recorded by oscilloscope. (**b**) Topolectrical equivalent circuit map of polyacetylene polymer.

The topolectrical circuit that is designed based on the SSH model, must have a row of capacitors (*i.e.* C_b_ and C_a_), which are separated from each other by inductors (*i.e.* L), and each of them is connected to the ground (Fig. 1c). When the C_b_/C_a_ ratio becomes less than one, a topological boundary mode appears^9^. Apparently, there must be a “fingerprint frequency”, in which formation of a topological boundary mode is expected. It might be where that the polyacetylene’s equivalent circuit has maximum conductivity. To assay the accuracy of the aforementioned conclusion, polyacetylene’s equivalent topolectrical circuit, was electrically stimulated with a voltage of 5 V_pp_ and the output was recorded with an oscilloscope (Fig. 4). It was observed that at a frequency of 373 kHz, both the amplitude and phase of the input and output modes were the same (Fig. 3a), which confirms that at certain frequencies the polyacetylene’s equivalent circuit has the absolute electrical conductivity, which are named as “fingerprint frequency” (Fig. 3b).

## Conclusion

In the present study, we studied the polyacetylene polymer lattice with two independent approaches (classical and quantum physics). Based on what is mentioned in the Eq. (1) and Supplementary information—Eqs. 6, 16, the variables λ, t and C were extracted, respectively. Aligning the aforementioned equations, one can find out that the λ, t and C variables are replaceable. Since, the t-parameter explains electron hopping in the quantum chain of the polyacetylene polymer, the other two parameters (i.e. λ and C) could give the same description in classical models. By these means, the polyacetylene’s equivalent topolectrical circuit, could be considered as an accurate classical model to study the electrical behavior of the polyacetylene quantum chain. This emulated and low-cost setup (*i.e.* polyacetylene’s equivalent topolectric circuit), not only enables us to study the electron quantum hopping in the polymer lattice on a macroscopic and laboratory scale, but also facilitates assay of other similar quantum phenomena in the same way. Further, the polyacetylene’s equivalent topoelectrical circuit, introduces promising approaches in molecular electronics and topological material studies.

## Methods

In Fig. 1, all the steps performed in the current study, from conceptual design to recording the results, are depicted. In the following sections, each of these steps is explained separately and in detail.

## Experiment

### Equivalent experimental electrical circuit

As we know, different physical systems show the same behavior if they are described by the same dynamic equations. Therefore, for a mechanical system, an equivalent electrical system (*e.g.* equivalent electrical circuit) could be simulated as well. Then, for a 1D electric circuit (Fig. 1, panels c and d), the network dispersion relation could be rewritten in Eq. (1), according to Supplementary information—Eq. (16):

1 $${\varepsilon }_{k}=\pm \sqrt{{C}_{a}^{2}+{C}_{b}^{2}+2{C}_{a}{C}_{b}Cos(2ak)}$$

where the coupling ${t}_{1}$ and ${t}_{2}$ coefficients are equivalent to C_b_ and C_a_ capacitors in the circuit (Eq. (2)), respectively (Fig. 1 panels c and d).

2 $$\frac{{t}_{1}}{{t}_{2}}=\sqrt{\frac{{C}_{b}}{{C}_{a}}}$$

The one-dimensional (1D) model exactly maps to the SSH model for the electronic excitations of the polyacetylene polymer $\left({\left({CH}_{2}\right)}_{n}\right)$, as a linear polymer with conjugated single and double bonds between carbon atoms.

The equivalent electrical circuit of polyacetylene is made of alternating capacitors (*i.e.* C_a_ and C_b_), among which there are a number of same inductors L, all of which are connected to the ground. The variable that is the index of topological edge formation in the circuit, is obtained from the C_a_/C_b_ ratio, in the condition that t < 1^9^. If the mentioned conditions are met and the polyacetylene’s equivalent circuit was electrically excited with a voltage of 5 V_pp_, it would be expected that the excitation and recording voltage ranges coincide in some frequencies, during mapping the recording spectrum. If the points of interest are matching at the exciting and recording voltages at different frequencies of the spectrum have the same phase, we can expect to see formation of an edge effect at the excitation frequency^9^. As shown in Fig. 1 – f, an electrical circuit that was previously designed based on the SSH model, is implemented on a printed circuit board. To build such a circuit, according to the Supplementary information—Eq. (17c), in which the numerical value of E_g_ was equal to 1.43 eV, the values of the variables ${t}_{1}$ and ${t}_{2}$ were adapted from the “Only ${t}_{1}$” column (Fig. 1i1). Accordingly, ${t}_{1}$ and ${t}_{2}$ will have values equal to 3.89 and 3.17 eV, respectively, and the ${t}_{1 }/{t}_{2}$ ratio will be equal to 0.82. Now, by using the Eq. (2), the numerical values of the capacitors C_a_ and C_b_ would be obtained equal to 0.33 and 0.22 micro-Farad, respectively; and the inductors (L) would be equal to 10 micro-Henry. Then, the topolectrical circuit would be arranged according to the aforementioned parameters^9^. In order to obtain the exact capacity that is expected from the capacitors and inductors used in the electrical circuit, it is necessary to test each one individually before placing them in the circuit arrangement. By this regard, all capacitors and inductors were placed in the circuit with 5% error. By connecting sockets at both ends of the circuit to the pins it was possible to have either the topological or non-topological circuits available, depending on the displacement of these connections (explained in Fig. 4—Panel b). In this regard, the electrical circuit was subjected to electrical stimulation by the function generator (GWINSTEK-MFG-2120MA) in the range of 300 to 900 kHz with 5 V_pp_, and the output of the circuit was measured by both the oscilloscope (GWINSTEK-GDS-1072B) and spectrum analyzer (Agilent-E4407B).

The spectrum analyzer sweeps the area of interest with optimized intervals and both the input and output signals would be demonstrated with normalized amplitude.

### Supplementary Information

Supplementary Information.

## Data Availability

The study did not report any data.
