# Supplementary material for: Topolectrical Circuit Correspondence Design of Polyacetylene
Source: Sci Rep. 2023 Nov 27;13:20847. doi: 10.1038/s41598-023-48278-z (PMC10681999; doi:10.1038/s41598-023-48278-z)
Supplement: Supplementary file 1 — Supplementary Information. [file 41598_2023_48278_MOESM1_ESM.docx]

**Supplementary information**

**Topolectrical Circuit Correspondence Design of Polyacetylene**

M.R. Albooyeh, A. Sadeghi and S.M. Mohseni

*Department of physics, Shahid Beheshti university, Tehran 19839, Iran*

**1. Theory**

If one could efficiently define unsophisticated basis sets for self-consistent DFT, equally accurate and reliable tight-binding parameters would be derivable for real solid-state applications.

*Foulkes, W. M. C. & Haydock, R. Tight-binding models and density-functional theory. Physical review B 39, 12520 (1989).*

**1-1. Formalism of classical systems with topological phase**

As shown in Figure 1 – Panel A, consider a set of point particles connected by massless rods and springs. Small rods of length r (Figure 1-A_3_) connect the particles to a large main rod, and they are free to rotate around the junctions in a two-dimensional plane. The points to which the rods are hinged are located on a one-dimensional grid with R distances. Adjacent particles are coupled through elastic springs. In this case, we have N particles and N-1 springs. The equilibrium length of the springs (which is the same amount among all springs), is chosen so that the system is in an equilibrium state as shown in Figure 1-A_3_, with the rods halfway between the top and bottom. In this case, the rods have small oscillations around their equilibrium position. In the following sections, we see that by choosing a specific length for the rods, this classical system can be placed in a topological phase; where the long-wavelength excitations of the system have a frequency gap. In this topological phase, the system will have edge modes (Figure 1-A_4_).

We show the elongation of the springs relative to the equilibrium state by $\delta\mathcal{l}_{i}$ and the mass displacement by $\delta\theta_{i}$ ^11^.

$\vec{e}=\left[ \begin{aligned} {\delta\mathcal{l}}_{1} \\ {\delta\mathcal{l}}_{2} \\ \vdots\\ {\delta\mathcal{l}}_{N} \end{aligned} \right]ˎ\vec{u}=\left[ \begin{aligned} {\delta\theta}_{1} \\ {\delta\theta}_{2} \\ \vdots\\ {\delta\theta}_{N} \end{aligned} \right]$ (1)

According to Figure 1-A_3_, the state of equilibrium is such that the rods are placed between the top and bottom and do not make an angle with the $\hat{y}$ direction. By changing the length of the spring, the balance state can be created in such a way that the rods are between the top and bottom, respectively, but still make the angle ($\bar{\theta}\neq0$) of the $\hat{y}$ direction. On the other hand, the network structure gives a linear relationship between the displacement of the particles and the tension of the springs as follows, which is given through the “non-square matrix Q”:

$$\vec{e}=Q^{T}\vec{u} (2)$$

In which

$$\lambda_{1}=\frac{r cos\bar{\theta}\left( R-2r sin\bar{\theta} \right)}{\sqrt{R^{2}+4r^{2}{cos}^{2}\bar{\theta}}} ˎ \lambda_{2}=\frac{r cos\bar{\theta}\left( -R-2r sin\bar{\theta} \right)}{\sqrt{R^{2}+4r^{2}{cos}^{2}\bar{\theta}}} (3)$$

As a result, the $Q^{T}$ matrix is written as follows

$Q_{(N-1)\times N}^{T}=\left[ \begin{aligned} \lambda_{2} \lambda_{1} 0 0 0 \cdots\\ 0 \lambda_{2} \lambda_{1} 0 0 \cdots\\ 0 0 \lambda_{2} \lambda_{1} 0 \cdots\\ \vdots\vdots\vdots\ddots\vdots\vdots\\ 0 0 0 \cdots\lambda_{2} \lambda_{1} \end{aligned} \right]$ (4)

And for the square matrix D=Q$Q^{T}$ we have

D= ${QQ}^{T}=\left[ \begin{aligned} \lambda_{2}^{2} \lambda_{1}\lambda_{2} 0 0 0 0\cdots\\ \lambda_{1}\lambda_{2} \lambda_{1}^{2}+\lambda_{2}^{2} \lambda_{1}\lambda_{2} 0 0 0 \cdots\\ 0 \lambda_{1}\lambda_{2} \lambda_{1}^{2}+\lambda_{2}^{2} \lambda_{1}\lambda_{2} 0 0 \cdots\\ \vdots\vdots\vdots\ddots\vdots\vdots\\ 0 0 0 \cdots{\lambda_{1}\lambda}_{2} \lambda_{1}^{2} \end{aligned} \right]$ (5)

So the dispersion relation for the infinite system is as follows

$$\omega^{2}=2\lambda_{1}\lambda_{2}coskR+\left( \lambda_{1}^{2}+\lambda_{1}^{2} \right)\Longrightarrow\omega=\pm\sqrt{{\lambda_{2}^{2}+\lambda}_{2}^{2}+2\lambda_{1}\lambda_{2}\mathrm{coskR}} (6)$$

Substituting $\lambda_{1}$ and $\lambda_{2}$ from equation (3) we have:

$$\omega^{2}=\frac{2r^{2}{cos}^{2}\bar{\theta}}{R^{2}+4r^{2}{cos}^{2}\bar{\theta}}\left( \left( R^{2}+4r^{2}{sin}^{2}\bar{\theta} \right)-\left( R^{2}-4r^{2}{sin}^{2}\bar{\theta} \right)cos(kR) \right) (7a)$$

$$\omega^{2}=\frac{2r^{2}{cos}^{2}\bar{\theta}}{R^{2}+4r^{2}{cos}^{2}\bar{\theta}}\left( 8{sin}^{2}\bar{\theta}+\left( \frac{3}{2}R^{2}+2r^{2}{sin}^{2}\bar{\theta} \right)\times\left( kR \right)^{2}+\cdots\right) (7b)$$

The dispersion relation obtained for the internal modes of the system has an interesting behavior regarding the $\bar{\theta}$ states ($\bar{\theta}=0$ and $\bar{\theta}\neq0$), besides near the center of the *Brillouin zone* (when $k=0$). In the case of $\bar{\theta}\neq0$, the frequency spectrum is gapped near the area of $k\sim0$. However, in case of $\bar{\theta}=0$, the system does not have a frequency gap, which result in two different topological modes (Figure 1-A4).

- - 1. **Edge modes**

The existence of a frequency gap in the spectrum of small oscillations of the system is directly correlated to the existence of edge modes. In the introduced “tensegrity” system, the edge modes are the ones that are defined as zero-oscillation modes.

As mentioned earlier, in topological states the θ is either more or less than zero. The existence of “edge states” could be differentiated from “bulk properties” in the topological phases of the system. The number of edge states of a finite system is directly correlated to the bulk properties of that system, when it is considered as infinite. In addition, the domains’ boundary in a one-dimensional system is important as well. Consider a situation that two subsystems with different topology (θ < 0ˎ θ > 0) are connected together. At the connection point of two subsystems (i.e. the M in Figure 1-A_3_), where the topology is changed, the closing of the frequency gap will be inevitable, so that we have a gap in M-1 and M+1, but there is a gap in the connection point (M). Therefore, there would be zero modes at point M that move freely. The movement of this boundary in the direction of the system, does not result in energy transfer. In fact, the phonon (heat) conductivity of this system takes place through the formation of inter-domain walls and their movement.

**2. Formalism of SSH model of polyacetylene polymer**

The SSH model has been proposed for polyacetylene (CH)_x_ polymer ^1^. In the mentioned model, there are three parameters (Table 1), are t_0_, k and α, which respectively represent atomic units of hopping, spring constant normalized by √Mass, and electron-phonon coupling ^20^.

**Table 1 - Values of atomic units of polyacetylene polymer.**

| **Parameters** | **Unit** |
| --- | --- |
| Mass of carbon atom (M) | 12 u = 1.66 × 10^-27^ kg |
| Electron hopping parameter (t_0_) | 2.5 eV |
| Spring constant parameter (k) | $1.75 eV/({uÅ}^{2})\cong{10}^{-5} atomic unit$ |
| Constant parameter of electron-phonon interaction (α) | $1.19 eV(\sqrt{u}Å)\cong9.62 atomic unit$ |

When we have a dimerized ground state then:

$u_{n}={(-1)}^{n}u^{0} ˎ u^{0}=0.042 Å$

$u_{n}={(-1)}^{n}u^{0} \Longrightarrow u_{0}=u^{0} ˎ u_{1}={-u}^{0} ˎ u_{2}=u^{0} ˎ u_{3}={-u}^{0} ˎ etc$ (8)

Where the $u^{0}$ is the minimum energy of the ground state and $u_{n}$ is the configuration coordinate for displacement the n^th^ CH group along the symmetry axis of the molecule (x direction). Besides, for the dimerized chain the $u_{n}=0$.

On the other hand, the numerical value of $t_{01}$ and $t_{12}$ hopping parameters can be obtained as follows

$t_{n ˎ n+1}=t_{0}-\alpha\left( u_{n}-u_{n+1} \right)$ (9)

$t_{01}=t^{0}-\alpha\left( u_{0}-u_{1} \right)=t^{0}-2\alpha u^{0}\equiv t_{1} "single" bond$ (9a)

$t_{12}=t^{0}-\alpha\left( u_{1}-u_{2} \right)=t^{0}+2\alpha u^{0}\equiv t_{2} "double" bond$ (9b)

Where $t_{1}$ and $t_{2}$ refers to single and double bonds (weak and strong coupling) (Figure 1-A_1_).

Therefore, the electron Hamiltonian can be written as follows

$H_{e}=-t_{1}C_{0}^{\dagger}C_{1}-t_{2}C_{1}^{\dagger}C_{2}-t_{1}C_{2}^{\dagger}C_{3}-t_{2}C_{3}^{\dagger}C_{4}+h.c$ (10)

So that:

$$C_{2n}=\frac{1}{\sqrt{L/2}}\sum_{k} e^{i(2\pi)a.k}C_{k}^{e} even (10a)$$

$$C_{2n+1}=\frac{1}{\sqrt{L/2}}\sum_{k} e^{i(2\pi)a.k}C_{k}^{o} odd (10b)$$

$$k=\frac{2\pi\mathcal{l}}{\left( \frac{L}{2} \right).2a}\mathcal{l=}0ˎ 1ˎ 2ˎ\ldots(10c)$$

As a result, the Hamiltonian in momentum space can be written as follows

$$H^{e}=-\left[ \sum_{n=0}^{\frac{L}{2}} t_{1}\left( C_{2n}^{\dagger}C_{2n+1} \right)+t_{2}\left( C_{2n+1}^{\dagger}C_{2n+2} \right) \right]+h.c. (11)$$

Where in

$$\sum_{n=0}^{\frac{L}{2}} C_{2n}^{\dagger}C_{2n+1}=\frac{1}{\frac{L}{2}} \sum_{\begin{matrix} k k^{ˊ} \\ n \end{matrix}} e^{-i(2n)a.k+i(2n)a.k^{ˊ}}C_{k}^{e^{\dagger}}C_{k^{ˊ}}^{o}$$

$=\frac{1}{\frac{L}{2}} \sum_{k k^{ˊ}} \sum_{n} e^{i(2n)a(k-k^{ˊ})}C_{k}^{e^{\dagger}}C_{k^{ˊ}}^{o}$ (11a)

And also

$$\sum_{n=0}^{\frac{L}{2}} C_{2n+1}^{\dagger}C_{2n+2}=\frac{1}{\frac{L}{2}} \sum_{\begin{matrix} k k^{ˊ} \\ n \end{matrix}} e^{-i(2n)a.k+i(2n+2)a.k^{ˊ}}\left( C_{k}^{o} \right)^{\dagger}C_{k^{ˊ}}^{e}$$

$=\sum_{k} e^{i2ak}\left( C_{k}^{o} \right)^{\dagger}C_{k}^{e}+e^{-i2ak}\left( C_{k}^{e} \right)^{\dagger}C_{k}^{o}$ (11b)

In momentum space we have

$$H^{e}=\sum_{k} \left( \begin{matrix} C_{k}^{\dagger_{e}} & C_{k}^{\dagger_{o}} \end{matrix} \right)\left( \begin{matrix} 0 & {-t}_{1}{-t}_{2}e^{-i2ak} \\ {-t}_{1}{-t}_{2}e^{i2ak} & 0 \end{matrix} \right)\left( \begin{matrix} C_{k}^{e} \\ C_{k}^{o} \end{matrix} \right) (12)$$

Assuming that

$H(k)=\left( \begin{matrix} 0 & {-t}_{1}{-t}_{2}e^{-i2ak} \\ {-t}_{1}{-t}_{2}e^{i2ak} & 0 \end{matrix} \right)$ (13)

And further, by considering $H(k)=H(k+\frac{\pi}{a}\mathcal{l)}$, we have

$H^{e}=\sum_{k} C^{\dagger}(k)H(k)C(k)$ (14)

As a result

$$\left( H(k)-\varepsilon\right)\psi_{k}=0 \psi_{k}=\left( \begin{matrix} C_{1} \\ C_{2} \end{matrix} \right) (15)$$

So

$$det \left( \begin{matrix} -\varepsilon& -\left( t_{1}+t_{2}e^{-i2ak} \right) \\ -\left( t_{1}+t_{2}e^{i2ak} \right) & -\varepsilon\end{matrix} \right)=0 \Longrightarrow\varepsilon^{2}-\left( t_{1}+t_{2}e^{-i2ak} \right)\left( t_{1}+t_{2}e^{i2ak} \right)=0$$

${\Longrightarrow\varepsilon}_{k}=\pm\sqrt{t_{1}^{2}+t_{2}^{2}+2t_{1}t_{2}Cos(2ak)}$ (16)

If $t_{1}=t_{2}=t_{0}$, then

$$\varepsilon_{k}=\pm2t_{0}Cos\left( ak \right) (16a)$$

By displacing the equations $t_{1}=t^{0}-2\alpha u$ and $t_{2}=t^{0}+2\alpha u$ in the equation (16), we have

$\varepsilon_{k}=\pm\sqrt{\left( t^{0}-2\alpha u \right)^{2}+\left( t^{0}+2\alpha u \right)^{2}+2\left( t^{0}-2\alpha u \right)\left( t^{0}+2\alpha u \right)Cos(2ak)}$

$$=\pm\sqrt{2t_{0}^{2}+8\alpha^{2}u^{2}+2\left( t_{0}^{2}-4\alpha^{2}u^{2} \right)Cos(2ak)}$$

$$=\pm\sqrt{2t_{0}^{2}\left( 1+Cos(2ak) \right)+8\alpha^{2}u^{2}\left( 1-Cos(2ak) \right)}$$

$=\pm\sqrt{4t_{0}^{2}{Cos}^{2}(ak)+16\alpha^{2}u^{2}{Sin}^{2}(ak)} \equiv\pm\sqrt{\left( \varepsilon_{k}^{0} \right)^{2}+\Delta_{k}^{2}}$ (17)

Where $\varepsilon_{k}^{0}$ and $\Delta_{k}$ are as follows

$\varepsilon_{k}^{0}=-2t_{0}Cos\left( ak \right)$ (17a)

$\Delta_{k}=4\alpha u Sin(ak)$ (17b)

By displacing the numerical values of two parameters (*i.e.* α and u) from Table 1 in equation (17b) ^21^, the energy gap can be obtained as follows

2Δ=8αu=1.4 eV (17c)

Equation (16) can also be deduced from the mass and spring model, which is explained in equation (6). Based on this, in addition to the electron, the equation (16) can be extended to other intermediate particles such as phonon, photon, etc.

**3. Hamiltonian matrix**

Consider a chain of atoms where electrons can jump between adjacent atomic positions. The simplest model we can design for a chain is based on electron movement between sites of same intensity. If we want to write the Hamiltonian matrix corresponding to this hopping matrix, we will have:


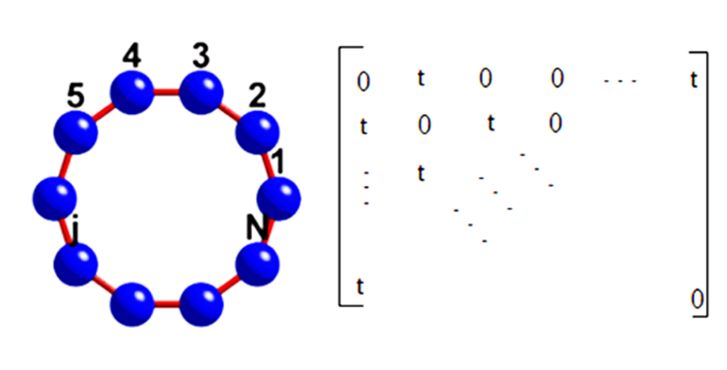


Figure 1: Hamiltonian matrix

As what is shown in Figure 1, there is no electron hopping from site-1 to itself. That’s why in the matrix, the (0, 1) array is registered as zero. However, there are transition from site-1 to 2 and also from site-1 to n. Therefore, in the first row of the matrix, except (0, 1) and (0, n) arrays, the rest of arrays in the first row of the matrix would be registered as zero.

Similarly, in the second row of the matrix, we have a hopping from site-2 to 1 and from site-2 to 3. So, we consider their related arrays in the matrix as t and the rest of the arrays in the second row of the matrix would be registered as zero.

If one continues to fill the arrays of the matrix in the same way, all the arrays above and below the main diameter, as well as the corners of the matrix, will be registered as t, while the rest of arrays would be Zero.

Then, Su and Scheriffer modified this model and assumed that if instead of registration of hopping as t everywhere, one would consider the arrays alternatively registered as t+Δ and t-Δ (Figure 2):


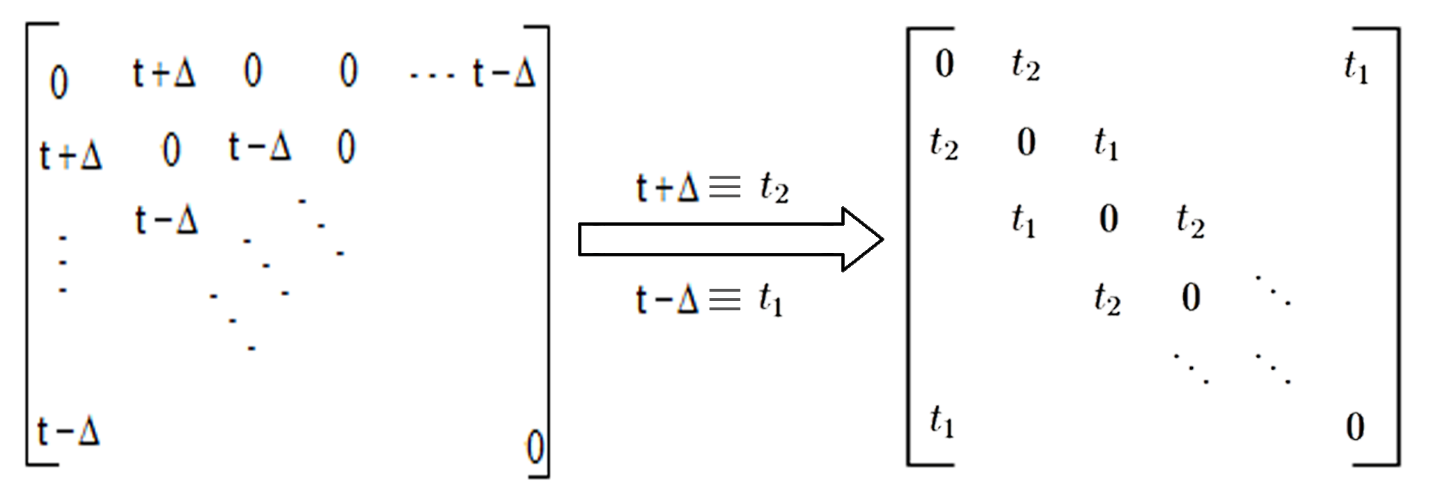


Figure 2: Hamiltonian matrix for periodic boundary condition.

As one can see in Figure 2, the left matrix changed to in such a way that when the hopping is made between sites 1 to 2 and between sites 2 to 1, the value of “t” turns to $t+\Delta$, while the hopping is made between sites 2 to 3 and between sites 3 to 2, the value of “t” turns to $t-\Delta$.

The system can be solved by Fourier transform, but in order to facilitate the calculation and provide a more accurate physical description, we consider two sites next to each other as a “unit cell”. With this trick, the system would become periodic. That means, if we go from one cell to another, the Hamiltonian does not change, because there will always be a hopping value (i.e. $t+\Delta$) when there is hopping from the first point to the second point. And there will always be a hopping value (i.e. $t-\Delta$), when there is a hopping from the second point to the third point. Of course, it should be noted that:

First: the network constant is 2a not a.

Second: The H matrix will not be diagonalized here, but it will be in the form of 2×2 blocks, and the sizes of momentums that are different by aa mount of “π” in size, could be combined with each other. It must be noted that the momentums were not allowed to be combined with each other, in the previous example.

The interesting point of the later example is that if we create a boundary for the system, the boundary border would be outlined as a matter of cutting the connection between site 1 and site n (i.e. there is no hopping from site 1 to site n). In this case, the two corners of the matrix would become zero, that is shown in Figure 3.


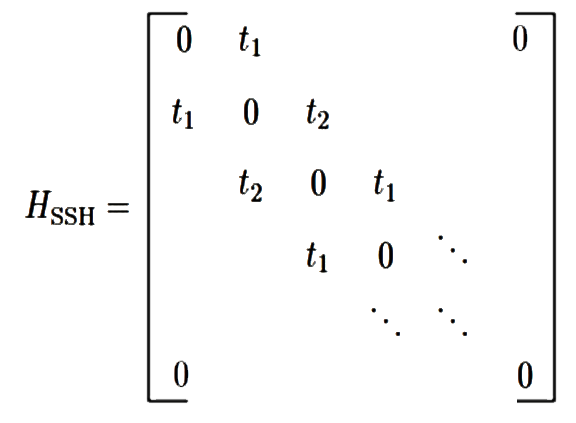


Figure 3: Hamiltonian matrix in SSH model for open boundary condition.

**4. Modeling and DFT calculations**

**4-1.Atomic structure:**

The infinite-length *trans*-polyacetylene configuration is modeled, as shown in Figure 1-A_1_, with repeating units of -HC=CH-, as unit cell in a one-dimensional periodic structure with a lattice constant of 2.45 Angstroms. In this structure, there are conjugated double and single carbon-carbon bonds, where the bond-lengths are 1.36 and 1.44 angstroms, respectively, while the angle between three consecutive carbon atoms is 122 degrees (Figure 1-A_2_) based on our calculations.

**4-2. The tight binding model:**

The tight-binding model provides an intuitive method to electron transfer process in the polyacetylene ^22,23^. Generally, the procedure consists of two steps:

1- Finding an analytic dispersion relation to describe the dependence of energy on electron momentum $\epsilon=\epsilon\left( \boldsymbol{k} \right)$ for the crystal of interest.

2- Fitting the parameters of the dispersion relation to either experimental data or first principle calculations.

The dispersion relation for the polyacetylene chain in the *trans* configuration, assuming that only the p_z_ orbitals of the carbon atoms have the possibility of leaving the original carbon atoms, includes two states: one occupied state and one unoccupied state. If the lattice constant of the one-dimensional polyacetylene chain is “a”, it can be shown that the dispersion relation reads:

$$\epsilon_{\pm}\left( k \right)= E_{F}\pm\sqrt{t_{1}^{2}+ t_{2}^{2}+ 2t_{1}t_{2}\cos k} (18)$$

The + sign, which gives rises to higher energy than the Fermi energy $E_{F}$, corresponds to the conduction band (unoccupied state) and the - sign corresponds to the valance band energy (occupied state). Note that the probability of electron hopping to the carbon atom with which it has a double bond (shorter bond length) is higher than the probability of hopping to the carbon atom on the other side with which it has a single bond (longer bond length). We show the two hopping parameters with $t_{1}$ and $t_{2}$, respectively. The smaller the difference between $t_{1}$ and $t_{2}$, the smaller the energy gap $E_{g}=\epsilon_{+}-\epsilon\_=2\left( t_{1}-t_{2} \right)$, while for the $t_{1}=t_{2}$ the chain is conductive i.e. with no gap.

**4-3. First-principles calculations:**

The calculations were done with Quantum Espresso software package. Since this software uses plane waves as basic functions for wave function expansion, it is suitable for the calculations of systems that are periodic in all three dimensions ^13^. In case of our chain of interest, which is periodic in only one dimension, the supercell method is applied where a vacuum space (with a width of more than 16 angstroms) is included in two directions perpendicular to the carbon chain, so that the periodicity of the chain in these two directions becomes negligible. Along the chain axis, the lattice constant “a” is 2.45 Å. The Cartesian coordinates of the atoms in the unit cell and the dimensions of the orthorhombic cell, shown in Figure 1 – panel A, are given in Table 2 (all lengths are in Angstroms). Parameters related to the coordinates of atoms in the cell structure (Figure 1-a_1_) are defined accordingly.

**4-4. Fitting the tight-binding parameters to the DFT results:**

As the last step, we fit the dispersion diagram resulting from the tight binding model (Equation 18), to the calculated data-points in the DFT framework, to determine the numerical value of the hopping parameters $t_{1}$ and $t_{2}$. Alternatively, one can consider the energy gap equal to the gap, predicted by DFT, and merely fit $t_{1}$ to the dispersion data, in which case we will have:

$t_{2}=t_{1}-E_{g}/2$ (19)
